# Tree species diversity promotes aboveground carbon storage through functional diversity and functional dominance

**DOI:** 10.1002/ece3.2525

**Published:** 2016-09-29

**Authors:** Sylvanus Mensah, Ruan Veldtman, Achille E. Assogbadjo, Romain Glèlè Kakaï, Thomas Seifert

**Affiliations:** ^1^ Department of Forest and Wood Science Stellenbosch University Matieland South Africa; ^2^ Laboratoire de Biomathématiques et d'Estimations Forestières Université d'Abomey‐Calavi Cotonou Bénin; ^3^ South African National Biodiversity Institute Kirstenbosch Research Centre Claremont South Africa; ^4^ Department of Conservation Ecology and Entomology Stellenbosch University Matieland South Africa; ^5^ Laboratoire d'Ecologie Appliquée Université d'Abomey‐Calavi Cotonou Bénin

**Keywords:** carbon stock, community weight mean, functional richness, maximum plant height, niche complementarity, structural equation modeling

## Abstract

The relationship between biodiversity and ecosystem function has increasingly been debated as the cornerstone of the processes behind ecosystem services delivery. Experimental and natural field‐based studies have come up with nonconsistent patterns of biodiversity–ecosystem function, supporting either niche complementarity or selection effects hypothesis. Here, we used aboveground carbon (AGC) storage as proxy for ecosystem function in a South African mistbelt forest, and analyzed its relationship with species diversity, through functional diversity and functional dominance. We hypothesized that (1) diversity influences AGC through functional diversity and functional dominance effects; and (2) effects of diversity on AGC would be greater for functional dominance than for functional diversity. Community weight mean (CWM) of functional traits (wood density, specific leaf area, and maximum plant height) were calculated to assess functional dominance (selection effects). As for functional diversity (complementarity effects), multitrait functional diversity indices were computed. The first hypothesis was tested using structural equation modeling. For the second hypothesis, effects of environmental variables such as slope and altitude were tested first, and separate linear mixed‐effects models were fitted afterward for functional diversity, functional dominance, and both. Results showed that AGC varied significantly along the slope gradient, with lower values at steeper sites. Species diversity (richness) had positive relationship with AGC, even when slope effects were considered. As predicted, diversity effects on AGC were mediated through functional diversity and functional dominance, suggesting that both the niche complementarity and the selection effects are not exclusively affecting carbon storage. However, the effects were greater for functional diversity than for functional dominance. Furthermore, functional dominance effects were strongly transmitted by CWM of maximum plant height, reflecting the importance of forest vertical stratification for diversity–carbon relationship. We therefore argue for stronger complementary effects that would be induced also by complementary light‐use efficiency of tree and species growing in the understory layer.

## 1 Introduction

The relationship between biodiversity and carbon storage is being debated as one of the current ecological topics (Cavanaugh et al., 2014; Day, Baldauf, Rutishauser, & Sunderland, 2014; Ruiz‐Benito et al., 2014; Ruiz‐Jaen & Potvin, 2011), and some aspects of climate‐related effects have been well investigated (Durán, Sánchez‐Azofeifa, Rios, & Gianoli, 2015; Wu et al., 2015). Because biomass is an important component of forest stand productivity, the relationship between biomass carbon and biodiversity can also be assimilated to the one of biodiversity and ecosystem function (Lasky et al., 2014). Basically, two well‐debated mechanisms are commonly used to explain the role of plant diversity in ecosystem resource dynamics, ecosystem processes, and functions: niche complementarity effects and selection effects (Díaz & Cabido, 2001; Tilman et al., 1997); the niche complementary effects hypothesis assumes increasing diversity would promote greater variety of functional traits and provide opportunities to species to efficiently use the available resources, thereby increasing ecosystem function; the selection effects hypothesis suggests that in ecosystem with higher diversity, there would be a higher probability of occurrence of dominant species or traits that influence ecosystem functioning. Currently, great research efforts are made to elucidate how diversity components (taxonomic diversity, functional diversity, and functional dominance) drive biomass and carbon stocks, and the extent to which the findings support niche complementarity and selection effects hypotheses.

Taxonomic diversity, expressed by species richness and alpha‐diversity indices, has been commonly used as a simple measure of biodiversity (Mayfield et al., 2010; Tilman et al., 1997) and has been shown to correlate positively with carbon stocks. However, because a new species—with different functional traits—added to an ecosystem would likely contribute to the physiological processes, the effects of taxonomic diversity on carbon storage could be treated as different effects of functional diversity (accounting for niche complementarity) or/and functional dominance (comprising selection effects). The functional diversity is known as “the value and range of functional traits of the organisms present in a given ecosystem”(Díaz & Cabido, 2001, pp 654) and therefore might be the starting point of elucidating the mechanisms underlying the relation between biodiversity and carbon (Cadotte, Carscadden, & Mirotchnick, 2011; Naeem, 2002). Yet, some recent reviews showed controversy in the relationship between taxonomic and functional diversity (Mayfield et al., 2010; Naeem, 2002; Song, Wang, Li, & Zhou, 2014). On the one hand, following Tilman et al. (1997) and Mouchet, Villéger, Mason, and Mouillot (2010), functional diversity was positively correlated with species richness, and in this case, taxonomic diversity can simply be used to replace functional diversity. On the other hand, it was pointed out that land use, the local species pool, etc. could also influence the relationship between functional and taxonomic diversity (Cadotte et al., 2011; Mayfield et al., 2010). Consequently, whether diversity (species richness) effects on ecosystem function are fully mediated by functional diversity or codetermined by selection effects (dominance patterns) is still well debated. In tropical natural forests, where several species cohabit and fulfill the major ecosystem functions, it is common to observe the abundance and dominance of highly productive tree species, thus increasing the chances that diversity–carbon relationships are mediated by selection effects. This was partly confirmed by our previous observations in South African mistbelt forests, especially the greater influence of the most dominant species on biomass stocks (Mensah, Veldtman, du Toit, Glèlè Kakaï, & Seifert, 2016). More and more, research tends to show how functional diversity and/or functional dominance play a major role in ecosystem functioning (Baraloto et al., 2012; Clark, Flynn, Butterfield, & Reich, 2012; Ruiz‐Jaen & Potvin, 2011; Song et al., 2014). Understanding whether diversity effects on ecosystem function are more likely mediated through functional diversity than functional dominance, or *vice versa*, will bring substantial insights into which mechanism is more relevant.

Very few studies have addressed the relationships between diversity and ecosystem function in natural multispecies tropical forests. Using aboveground tree carbon data in a northern mistbelt forest in South Africa, we examined the relationship between diversity and carbon stocks through the effects of functional diversity and functional dominance. We hypothesized that (1) diversity influences tree carbon storage through both functional diversity and functional dominance effects. However, there are insights that diversity and carbon relationships can be caused by covarying environmental factors (Cavanaugh et al., 2014; Ouyang et al., 2016). Therefore, we considered altitude and slope as the most physical gradients in these forests, and tested their effects on tree carbon storage. In addition, while accounting for significant environmental gradient effects, we also hypothesized that (2) effects of diversity on carbon storage would be greater for functional dominance than for functional diversity.

## 2 Materials and Method

### 2.1 Study area

This study was carried out in the northern mistbelt forests in the Limpopo Province, South Africa. These forests are generally found as large patches on steep eastern slopes in the province (Geldenhuys, 1997, 2002). The site selected for this study was the Woodbush‐De Hoek native forest complex (23°50′S, 30°03′E) near Tzaneen. The area is characterized by an altitudinal gradient from 1,050 to 1,800 m above mean sea level and an annual rainfall ranging from 600 mm to 1,800 mm (Geldenhuys, 2002). Pine plantations are established in the surrounding environment by the State Department of Water Affairs and Forestry and transferred to forest companies for commercial timber production. The main sectors for the management policy in the landscape are timber production, nature conservation, and recreation (hiking). The vegetation in the Woodbush‐De Hoek native forest is dominated by canopy and above canopy species such as *Xymalos monospora*,* Podocarpus latifolius*,* Syzygium gerrardii,* and *Cryptocarya transvaalensis* (Mensah, Glèlè Kakaï, & Seifert, 2016). The understory vegetation is represented by species such as *Oxyanthus speciosus*,* Peddiea africana*, and *Kraussia floribunda* (Geldenhuys, 1997).

### 2.2 Forest sampling and aboveground carbon data

Stand data (species, canopy layer, tree density, basal area) were obtained by means of a stratified random sampling design set in a 707.612 ha (hectare) forest block in the Woodbush‐De Hoek forest. The sampling design consisted of 30 replicates of 250 m^2^ circular subplots, each inside a 500 m^2^ circular larger plot. These plots were laid out in stratified compartments obtained by subdividing the research area on the basis of three classes of slope: flat (1.5%–15.3%), gentle (15.3%–29.19%), and steep (29.19%–43.1%); four classes of aspect (North, South, West, and East); and three classes of elevation: low (1,174–1,332 m a.s.l.), medium (1,332–1,490 m), and high (1,490–1,648 m). Inside 250 m^2^ plots, species names, diameter at breast height (dbh), and height of trees belonging to 5–10 cm dbh were recorded, while only individuals having more than 10 cm dbh were tagged and measured within the larger plots.

We used the multispecies allometric biomass equation developed for the northern mistbelt forests (Mensah, Veldtman, du Toit, Glèlè Kakaï, & Seifert, 2016) to calculate the aboveground biomass (AGB) for all individual trees present in the plots. The allometric equation provided more accurate estimated biomass values, compared with the existing pantropical biomass equation (Chave et al., 2005; Mensah, Veldtman, & Seifert, 2016). The formula for the allometric biomass equation is as follows:

$$\text{AGB}\;=\;1.03\;\times \;\text{exp}(-2.69\;+\;0.69\cdot \text{ln}\left(\text{SWD}\right)\;+\;0.95\cdot \text{ln}\left({\text{DBH}}^{2}\cdot H\right))$$

where AGB stands for the aboveground tree biomass in kilograms, SWD the specific wood density (g/cm^3^), DBH the diameter at breast height (cm), and *H* the total height (m). AGB was computed for each individual tree, upscaled to plot and stand level for each diameter class (i.e., for 5–10 cm dbh in the 30 smaller plots and for ≥10 cm dbh in the 30 larger plots), and summed up to obtain the values for dbh > 5cm. Carbon values were determined afterward, by multiplying the aboveground biomass by a factor of 0.5 (Lung & Espira, 2015).

### 2.3 Diversity and dominance metrics

Diversity was measured using taxonomic diversity, at each plot. We used species richness to characterize the taxonomic diversity (Magurran, 1988). Species richness at plot level is simply defined as the number of distinct species enumerated inside each plot. To assess functional diversity, we considered the functional traits that are relevant to the ecosystem function of interest (i.e., biomass and carbon storage). Because carbon storage is strongly dependent on wood and foliage structures, we used traits such as specific wood density (WD), specific leaf area (SLA), and maximum plant height (PHm). Data on specific wood density were obtained from the Global Wood Density Database (Zanne et al., 2009). In case multiple values were available for a single species, the average wood density was used. When a particular species was missing, we used the average genus wood density. SLA and PHm were extracted from the TRY database (Kattge et al., 2011). As functional diversity metrics, we estimated functional richness (Fric), functional evenness (Feve), functional divergence (Fdiv), functional dispersion (Fdis), and Rao quadratic entropy (RaoQ) at each plot (Baraloto et al., 2012; Cavanaugh et al., 2014; Finegan et al., 2015; Villéger, Mason, & Mouillot, 2008), using the values of the functional traits with the “FD” package in R (Laliberté, Legendre, & Shipley, 2015). These diversity indices are multitrait functional diversity metrics that combine both the relative weight of each species and the pairwise functional difference between species. A review of these measures can be found in study by Mouchet et al. (2010).

Functional dominance was assessed by estimating the plot‐level community weight mean (CWM) for each functional trait. CWM is the mean of each species trait value weighted by the relative abundance (stem number) of that species (Cavanaugh et al., 2014). The per‐plot CWM was estimated for WD, SLA, and PHm, again using the “FD” package in R.

### 2.4 Data analyses

Here, we tested two hypotheses: (1) diversity effects on carbon storage are mediated through both functional diversity and functional dominance effects; and (2) effects of diversity on carbon storage are greater for functional dominance than for functional diversity. The first hypothesis was tested using structural equation models (SEM), while the second hypothesis was tested using linear mixed‐effects models. For both SEM and linear mixed‐effects models, AGC data were log‐transformed to meet the normality assumption (Shapiro–Wilk statistic = 0.97, *p*‐value = .628).

#### 2.4.1 Structural equation modeling

SEM offers the possibility to test hypothesized patterns of direct and indirect relationships among the measured variables. This is particularly important, as we assumed that the diversity effects would be transmitted through both functional diversity and functional dominance. Therefore, we examined the indirect and direct effects of diversity (species richness) on aboveground carbon. We constructed two separate structural equation models representing (1) full mediation: Diversity effects are fully transmitted by functional diversity and dominance metrics; and (2) partial mediation: There are both direct and indirect diversity effects through functional diversity and functional dominance metrics. Due to the presence of multiple measures for functional diversity, we used stepwise selection techniques to select the most relevant functional diversity metrics for the aboveground carbon data. As a result, only functional richness (Fric) and functional evenness (Feve) were selected (*p*‐value <.05). We did not deny the potential effects of environmental variables on the species diversity and aboveground tree carbon. Nevertheless, we believe that such effects could be better assessed in mixed modeling approach (addressed in the next paragraph), thus simplifying the outputs of the SEMs. The overall fit of the SEMs was assessed using χ^2^ – square test (a *p*‐value >.05 would indicate an absence of significant deviations between data and model), the comparative fit index (CFI), and the Akaike information criterion (AIC) (Grace & Bollen, 2005). We used the standardized coefficients to allow direct comparisons across paths (Grace & Bollen, 2005). SEMs were fitted in the R statistical software package (R Development Core Team 2015), using the “sem” functions in the “lavaan” package version 0.5–19 (Rosseel, 2012).

#### 2.4.2 Linear mixed‐effects models

Prior to the mixed‐effects modeling, we tested for potential environmental variables and species richness effects on aboveground carbon storage. Environmental variables are expected to have effects on plant structures, growth, and survival (Mensah, Houehanou, Sogbohossou, Assogbadjo, & Glèlè Kakaï, 2014; Wang, Fang, Tang, & Zhu, 2006) and hence on standing aboveground biomass and carbon stocks. Here, we focused on the variables that are determinant and quantifiable in the area, that is, topography (slope and altitude) (Geldenhuys, 1997, 2002). Other environmental variables such as temperature and precipitation have also been proved to have much influence on productivity, biomass, and carbon stocks (Cavanaugh et al., 2014; Durán et al., 2015), but were not explored here mainly because of their unavailability at the small scale in this study. Topography was characterized by classifying the slope and elevation in three categorical levels. Slope was categorized as flat (low slope), gentle (intermediate slope), and steep (high slope). As for elevation, low, medium, and high categories were considered. Simple linear models were performed to test for slope and elevation effects on aboveground carbon storage. As a result, only the slope showed slightly significant impact on the carbon stock and therefore was considered for further analyses. Multiple linear regressions were also fitted on slope and species richness to test their effects on aboveground carbon storage. For both simple and multiple linear models, Shapiro–Wilk tests were used to check for the normality of the log‐transformed AGC data and of the residuals. Additionally, Breusch–Pagan tests and Durbin–Watson statistics were used to test for homoscedasticity and autocorrelation between residuals, respectively.

We next examined the relationship of each diversity component (i.e., functional diversity and functional dominance) with carbon storage, by fitting separate linear mixed‐effects models (Zuur, Ieno, Walker, Saveliev, & Smith, 2009). We considered species richness and slope as random factors, and each measure of functional diversity (i.e., Fric, Feve, Fdiv, Fdis, and RaoQ) and of functional dominance (i.e., CWM of WD, SLA, and PHm) as fixed effects. The mixed‐effects models were fitted to assess (1) individual effect of each functional diversity and functional dominance metric; (2) combined effects of functional diversity metrics; (3) combined effects of functional dominance metrics; and (4) combined effects of functional diversity and functional dominance metrics. The best models were selected by performing a backward elimination of nonsignificant effects (*p*‐value >.05). The linear mixed‐effects models were performed using the “lmer” function of the “lmerTest” package (Kuznetsova, Brockhoff, & Christensen, 2016) of the R statistical software. The *p*‐values reported were calculated from the *F* test based on Satterthwaite approximations to the degrees of freedom, in the package “lmerTest” (Kuznetsova et al., 2016). The significance of the random effects was assessed using likelihood ratio (LR) test, again in the package “lmerTest”. The performance of fitted models was assessed based on the fit statistics such as Akaike information criterion (AIC) and the marginal R square, which indicates the proportion of variance explained by fixed effects (Nakagawa & Schielzeth, 2013).

## 3 Results

A total of 50 plant species were enumerated, belonging to 46 genera and 33 families. The dominant families were Rutaceae (five species), Rubiaceae (four species), Stilbaceae (three species), and Celastraceae (three species). The number of species per plot, for trees ≥5 cm dbh, ranged from five species to 18 species, with an average species richness of 11 species per plot. Tree number varied from 19 to 67 stems, with an average value of 42 stems per plot. The amount of aboveground carbon was highly variable across all plots, and ranged from 49.1 MgC/ha to 476.1 MgC/ha, with an estimated average value of 179 MgC/ha.

### 3.1 Diversity effects mediated through functional diversity and functional dominance

**Figure 1:**
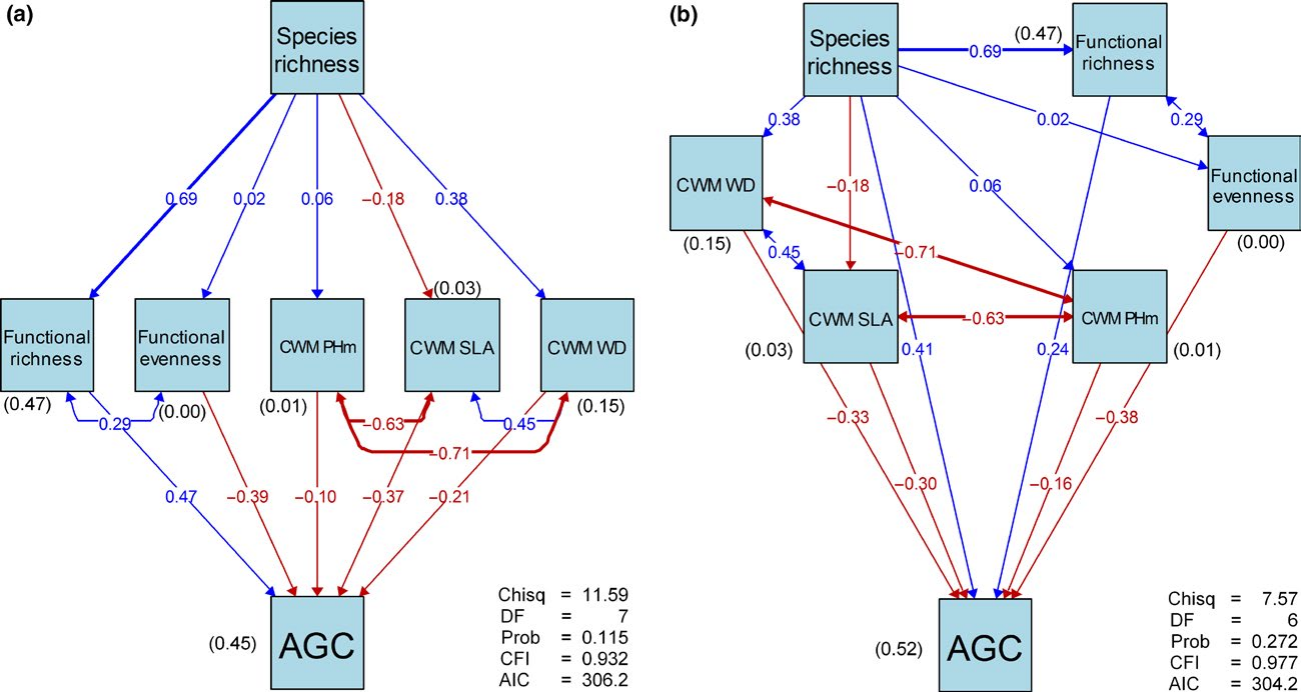
Summary of the path model relating species diversity (species richness), and measures of functional diversity and of functional dominance to the aboveground carbon (AGC); a: full mediation; b: partial mediation. CWM: community weight mean; PHm: maximum plant height; SLA: specific leaf area; WD: wood density. The figures with parentheses are the coefficients of determination (*R* ^2^), shown for dependent variables. The figures without parentheses are the standardized path coefficients. The single‐pointed arrows represent causal paths, while the double‐pointed arrows represent the residual correlations. The blue lines indicate the positive effects, while the red lines show negative effects; Chisq, Chi‐square statistic; DF, degree of freedom indicating the number of paths omitted from the model; Prob, probability of the data given the model; Prob >.05 indicates the absence of significant discrepancy between the data and the model. CFI, comparative fit index; AIC, Akaike information criterion. The significance of each path is given in Table 1

The outputs of the structural equation models fitted to assess the mediated effects of diversity (species richness) on AGC, through functional diversity and functional dominance, are summarized in Table 1 and Figure 1. The first model “full mediation” had chi‐square value of 11.59 (df = 7; *p* = .115), indicating good fit and absence of significant deviations between data and model.

**Table 1:** Results of the structural equation modeling carried out to test the effects of species richness on carbon stocks (AGC) via functional diversity and functional dominance

	Est.std	*SE*	Z	*p*‐value	Est.std	*SE*	Z	*p*‐value
	Full mediation				Partial mediation			
Regressions								
Path from species richness to Fric	0.69	0.14	5.02	<.001	0.69	0.14	5.02	<.001
Path from species richness to Feve	0.02	0.19	0.09	.926	0.02	0.19	0.09	.926
Path from species richness to CWM (PHm)	0.06	0.19	0.32	.750	0.06	0.19	0.32	.750
Path from species richness to CWM (SLA)	−0.18	0.19	−0.99	.324	−0.18	0.19	−0.99	.324
Path from species richness to CWM (WD)	0.38	0.18	2.20	.028	0.38	0.18	2.20	.028
Path from Fric to AGC	0.47	0.16	3.04	.002	0.24	0.19	1.27	.203
Path from Feve to AGC	−0.39	0.14	−2.70	.007	−0.38	0.14	−2.75	.006
Path from CWM (PHm) to AGC	−0.10	0.22	−0.46	.642	−0.16	0.21	−0.77	.440
Path from CWM (SLA) to AGC	−0.37	0.18	−2.06	.039	−0.30	0.17	−1.74	.081
Path from CWM (WD) to AGC	−0.21	0.19	−1.09	.275	−0.33	0.20	−1.66	.096
Path from species richness to AGC					0.41	0.20	2.00	.046
Residual correlations								
Path from CWM (WD) to CWM (SLA)	0.45	0.15	3.02	.003	0.45	0.15	3.02	.003
Path from CWM (WD) to CWM (PHm)	−0.71	0.09	−7.50	<.001	−0.71	0.09	−7.50	<.001
Path from CWM (SLA) to CWM (PHm)	−0.63	0.11	−5.54	<.001	−0.63	0.11	−5.54	<.001
Path from Feve to Fric	0.29	0.17	1.69	.090	0.29	0.17	1.69	.090
Model fit statistics								
AIC	306.2				304.2			
*p*‐value (chi‐square)	.115				.275			
*R* ^2^	0.45				0.52			

Est.std, path standardized coefficients; *SE*, standard error; Fric, functional richness; Feve, functional evenness; CWM, community weight mean; PHm, maximum plant height; SLA, specific leaf area; WD, wood density.

In the “full mediation” model, species richness showed a significant positive direct effect on functional richness (*R* ^2^ = 0.47; β = 0.69; *p *<* *.001; Table 1), which also showed positive and significant effect on AGC (β = 0.47; *p *=* *.002; Table 1). Therefore, species richness, through functional richness, had a positive significant effect on AGC (β = 0.69*0.47 = 0.32). There was a nonsignificant effect of species richness on functional evenness (β = 0.02; *p *=* *.926; Table 1); the latter, however, exhibited a significant negative effect on AGC. In addition, we found no significant correlation between functional richness and functional evenness (β = 0.29; *p *=* *.090; Table 1), which would suggest that the mediated effects of species richness were transmitted by functional richness only. Among the functional dominance metrics, the CWM of maximum plant height did not retain any significant path. Only the CWM of wood density showed significant responses to species richness (*R* ^2^ = 0.15; β = 0.38; *p *=* *.028), but did not significantly influence the AGC (*p *=* *.275). In contrast, the CWM of SLA had a negative significant effect (β = −0.37; *p *=* *.039; Table 1) on AGC, although not significantly influenced by species richness (*p *=* *.324). The significant residual correlation between CWM of wood density and CWM of SLA (β = 0.45; *p *=* *.003; Table 1) suggests that the mediated effects of species richness are also transmitted by these two factors.

Note that the “partial mediation” model was fitted by only adding a direct path from species richness to AGC to the “full mediation” model. The chi‐square value for the “partial mediation” model was 7.57 with 6 degrees of freedom and a *p* value of .272, also indicating good fit. There are similarities between the two models in terms of significant and nonsignificant paths (Table 1), but the “partial mediation” model exhibited slightly better fits (CFI = 0.932; *R* ^2^ = 0.52; AIC = 304.2) than the “full mediation” model (CFI = 0.977; *R* ^2^ = 0.45; AIC = 306.2). The added causal path from species richness to AGC was slightly significant at 0.05, suggesting an existing true direct effect of diversity on AGC. Both models suggest that species richness effects on aboveground carbon are mediated through functional diversity and functional dominance.

### 3.2 Effects of environmental variables, functional diversity, and functional dominance on carbon storage

Not surprisingly, there were significant effects of the environmental variables, especially the slope which explained 14.05% of the variation of the aboveground carbon (Table 2). Low slope showed regression coefficient which was 0.53 significantly higher than the baseline (higher slope), whereas intermediate slope was not. This indicates that carbon stock was significantly higher at low slope sites than high slope sites. Unlike slope, altitude did not have any significant influence on the aboveground tree carbon (*F*‐statistic = 1.381; *p *=* *.268; Table 2). Furthermore, while accounting for the effects of the slope, we also found that species richness was significant and showed a positive relationship with AGC (β = 0.06; *p *=* *.016; Table 2).

**Table 2:** Results of simple and multiple linear models testing the effects of elevation, slope, and richness on aboveground carbon stock

		Est.	*SE*	*t* value	Pr (>|*t*|)	SW	BP	DW
Elevation	(Intercept)	12.15	0.19	63.48	<0.001	0.849	0.240	1.68
	Low	−0.36	0.24	−1.48	0.152			
	Medium	−0.09	0.23	−0.40	0.691			
	Adjusted *R* ^2^ (%)	2.56						
Slope	(Intercept)	11.67	0.20	59.24	<0.001	0.927	0.211	1.69
	Low	0.53	0.23	2.32	0.028			
	Medium	0.19	0.24	0.84	0.409			
	Adjusted *R* ^2^ (%)	14.05						
Slope + Species richness	(Intercept)	10.98	0.32	34.19	<0.001	0.821	0.263	1.93
	Low	0.51	0.21	2.45	0.021			
	Medium	0.16	0.22	0.72	0.479			
	Species richness	0.06	0.03	2.56	0.017			
	Adjusted *R* ^2^ (%)	28.71						

Est., estimates of regression coefficients; *SE*, standard errors; SW, *p*‐values for Shapiro–Wilk normality tests; BP, *p*‐values for Breusch–Pagan tests; DW, Durbin–Watson autocorrelation statistic.

The results of the separate linear mixed‐effects models testing the individual effects of functional diversity metrics revealed that only Feve was significant, and had a negative effect on AGC (β = −1.6; *p *=* *.037; Table 3). Fdis, Fdiv, and RaoQ showed high values of probability (from 0.359 to 0.528), while Fric had a slightly significant and positive effect on AGC (*p *=* *.079; Table 3). While assessing the combined effects of functional diversity metrics, we found that Fdis, Fdiv, and RaoQ were left out after backward selection for the final model (Table 3). The effects of functional diversity on AGC were thus shown by a significant positive effect of functional richness (β = 135.6; *p *=* *.013; Table 3) and a significant negative effect of functional evenness (β = −2.03; *p *=* *.006; Table 3). Both functional richness and evenness explained 27% of the variance of AGC.

**Table 3:** Results of linear mixed‐effects models testing the effects of functional diversity on aboveground carbon stock

	Fixed effects					Random effects (variance)				
	Est.	*SE*	df	*t*	Pr (>|*t*|)	Sp.rich.	Slope	Rsd.	Marg. *R* ^2^	AIC
(Intercept)	11.76	0.16	2.98	71.90	<0.001	0.00	0.05	0.15	0.09	30.74
Fric	103.06	56.38	24.19	1.83	0.079					
(Intercept)	12.92	0.48	25.97	27.11	<0.001	0.00	0.03	0.15	0.13	37.96
Feve	−1.66	0.75	24.58	−2.21	0.037					
(Intercept)	11.75	0.27	8.16	43.48	<0.001	0.00	0.05	0.17	0.01	40.77
Fdis	1.00	1.57	25.82	0.64	0.528					
(Intercept)	12.30	0.446	22.51	27.577	<0.001	0.01	0.02	0.16	0.03	41.95
Fdiv	−0.64	0.686	25.47	−0.935	0.359					
(Intercept)	11.77	0.22	4.18	53.14	<0.001	0.00	0.06	0.17	0.02	38.38
RaoQ	3.82	4.66	25.80	0.82	0.42					
(Intercept)	12.97	0.43	24.83	30.08	<0.001	0.00	0.04	0.12	0.27	23.83
Fric	135.59	50.64	23.15	2.68	0.013					
Feve	−2.03	0.68	23.32	−2.97	0.006					

Est., coefficient estimates; *SE*, standard errors; Sp.rich., species richness; Rsd., residual variance; Marg. *R* ^2^, marginal R square; Fric, functional richness; Feve, functional evenness; Fdis, functional dispersion; Fdiv, functional divergence; RaoQ, Rao quadratic entropy.

All the three functional dominance metrics used in this study showed significant effects on the aboveground carbon (Table 4). Both CWM of SLA and CWM of WD showed negative effects, while CWM of maximum plant height exhibited a positive effect (Table 4). However, when assessing their combined effects on AGC, CWM of SLA was not retained by the final model, and the effects of functional dominance were only shown by positive and significant effects of CWM of maximum plant height and CWM of wood density, with 21% explained variance (Table 4).

**Table 4:** Results of linear mixed‐effects models testing the effects of functional dominance on aboveground carbon stock

	Fixed effects					Random effects (variance)				
	Est.	*SE*	df	*t*	Pr (>|*t*|)	Sp.rich.	Slope	Rsd.	Marg. *R* ^2^	AIC
(Intercept)	13.92	0.66	18.99	21.15	<0.001	0.03	0.03	0.10	0.20	44.18
CWM (SLA)	−0.02	0.01	17.55	−3.14	0.006					
(Intercept)	10.21	0.51	20.18	20.14	<0.001	0.08	0.11**	0.07	0.17	41.29
CWM (PHm)	0.07	0.02	18.45	3.66	0.002					
(Intercept)	14.85	1.19	16.42	12.46	<0.001	0.15	0.05	0.09	0.10	38.39
CWM (WD)	−4.86	1.94	15.37	−2.50	0.024					
(Intercept)	6.06	2.06	24.64	2.95	0.007	0.00	0.16**	0.11	0.21	38.03
CWM (PHm)	0.11	0.03	24.35	3.63	0.001					
CWM (WD)	5.35	2.44	23.96	2.19	0.038					

**Significant at 0.01.

Est., coefficient estimates; *SE*, standard errors; Sp.rich., species richness; Rsd., residual variance; Marg. *R* ^2^, marginal R square; CWM (SLA), community weight mean of specific leaf area; CWM (WD), community weight mean of wood density; CWM (PHm), community weight mean of maximum plant height.

Examination of separate mixed‐effects models for functional diversity and functional dominance revealed that the marginal *R* square (variance explained by fixed factors) in the diversity–AGC relationship was greater for functional diversity (27%) than for functional dominance (21%). When considering functional diversity and functional dominance measures in a same model, we found that 34% of the variations of AGC were explained by significant effects of functional richness, functional evenness, and CWM of maximum plant height (Table 5). For all the selected models, species richness as random factor had much less variability than slope. The nonsignificant variability due to species richness in the mixed‐effects models suggests that much of its influence on AGC has been considered by functional diversity and functional dominance, as confirmed by the SEM.

**Table 5:** Results of linear mixed‐effects models testing the combined effects of functional diversity and functional dominance on aboveground carbon (AGC) stock

Model description			Est.	*SE*	df	*t*	Pr (>|*t*|)
Functional diversity + Functional dominance	Fixed effects	(Intercept)	11.39	0.63	23.82	18.03	<0.001
		Fric	143.50	42.65	21.99	3.37	0.003
		Feve	−1.72	0.58	22.15	−2.95	0.008
		CWM (PHm)	0.06	0.02	22.80	3.10	0.005
	Random effects (variance)	Species richness	0.00				
		Slope**	0.09				
		Residual	0.08				
		Marginal *R* ^2^	0.34				
		AIC	24.28				

**Significant at 0.01.

Est., coefficient estimates; *SE*, standard errors; Fric, functional richness; Feve, functional evenness; CWM (PHm), community weight mean of maximum plant height.

## 4 Discussion

Our study explored the patterns of diversity–carbon stock relationship in mistbelt forests in South Africa, finding that carbon stocks varied greatly as responses to environmental gradients, taxonomic diversity, functional diversity, and functional dominance. Specifically, the study revealed that (1) slope gradient significantly influenced aboveground carbon, with lower carbon stock found at steeper sites; (2) increasing species diversity (species richness) increased tree carbon stock; (3) diversity effects on tree carbon stock were mediated through functional diversity and functional dominance; (4) functional diversity effects on tree carbon stock were greater than those of functional dominance; and (5) the specific effects of functional diversity and functional dominance on carbon stock varied with metrics and functional traits.

### 4.1 Effects of environmental variables on tree carbon storage

We did not detect any significant effect of altitude on tree carbon stock, according to Cavanaugh et al. (2014) who also reported in a global scale study, a lack of significant relationship between forest carbon and altitude. Yet, this finding runs contrary to many previous studies that examined the relationships between altitude and biomass or carbon storage (de Castilho et al., 2006; Ensslin et al., 2015; Sharma, Baduni, Gairola, Ghildiyal, & Suyal, 2010). On the one hand, some authors reported that biomass and carbon stocks can decline with increasing altitude (de Castilho et al., 2006; Moser, Hertel, & Leuschner, 2007). On the other hand, studies found positive correlation between increasing tree carbon and increasing altitude (Gairola, Sharma, Ghildiyal, & Suyal, 2011; Zhu et al., 2010). Furthermore, biomass and carbon stocks were found to increase up to a certain altitudinal limit (3,000 m a.s.l.) and afterward decline sharply with higher altitudinal values (Ensslin et al., 2015; Singh, Adhikari, & Zobel, 1994). This lack of clarity on the relationship between altitude and forest biomass may be partly due to the variation in the altitudinal range among studies. For instance, most of the abovementioned studies that reported significant effects of altitude have covered greater altitudinal ranges well above 2,500 m a.s.l; the relationship between altitude and carbon stocks in our study might have been hidden due to the smaller altitudinal range covered (1,000–1,800 mm), which might have not been considerable enough to detect substantial variation in growth conditions and hence biomass and carbon stock.

Unlike altitude, slope showed significant influence, and accounted for 14% of carbon variance, evidencing that differences in carbon stocks can result from topological constraints, particularly difference in slope. Consistent with our results, slope has been identified as a potential environmental variable that affects tree carbon (de Castilho et al., 2006; Chave et al., 2003). Because aboveground carbon is intrinsically related to wood and biomass production, the influence of slope can be seen as prior impacts of environment on availability of resources (de Castilho et al., 2006; Luizao et al., 2004), which in turn affect forest dynamics. For example, steeper slope will speed up nutrients and water runoff and constrain trees and will also favor highly water and nutrient efficient species against others. Taking this into account, it follows that tree growth and biomass production can be potentially reduced at higher slope sites, as results of moisture and nutrient stress (Clark, Clark, & Oberbauer, 2010; Durán et al., 2015), whereas flat and gentle slope sites would allow for more water availability, to which plant would likely respond positively. The significant effect of slope supports the fact that ecosystem functions in general and biomass and carbon storage in particular are environment‐structured (Wu et al., 2015).

### 4.2 Increasing species diversity promotes tree carbon storage

We found significant and positive effects of species richness on aboveground carbon, even when the effects of environmental factors (i.e., slope) were accounted for. While this finding accords with some recent studies that controlled for the effects of environmental variables (Ouyang et al., 2016; Wu et al., 2015), it also supports the commonly described pattern in highly diverse natural forests; that is, biomass and carbon stocks increase with increasing diversity. Indeed, several local and global studies on forest ecosystems have shown positive relationship between species richness and forest biomass or carbon (Cavanaugh et al., 2014; Con et al., 2013; Day et al., 2014; Ruiz‐Benito et al., 2014; Sharma et al., 2010; Wu et al., 2015). In addition, studies in boreal (Paquette & Messier, 2011), temperate (Paquette & Messier, 2011; Vilà et al., 2007), and tropical forests (Barrufol et al., 2013) have also reported increases in productivity with increasing diversity.

One can expect that increasing species diversity would increase carbon storage because higher taxonomic diversity would lead to higher stem density and forest productivity (Ruiz‐Benito et al., 2014). The positive effect of species diversity can also be explained through the benefits of plant–plant interactions such as facilitation, where by some species could enhance soil fertility (by fixing nitrogen) for the productivity of other species. This fact is even often used to support the reason why mixed species communities of plantations are far more productive than monospecific stands. But it might also be well possible that increasing species richness increases the chances of inclusion of highly productive and naturally favored dominant species (Ruiz‐Benito et al., 2014), as shown by our previous results on the influence of most dominant species on carbon stocks in mistbelt forests (Mensah, Veldtman, du Toit, Glèlè Kakaï, & Seifert, 2016).

While our dataset in the mistbelt forests supports the positive species richness–carbon relationship, it must be noted that evidence of the inverse effect also exists. For instance, studies by Ruiz‐Jaen and Potvin (2011) in natural forest of Barro Colorado Island in Central Panama and Szwagrzyk and Gazda (2007) in natural forests of central Europe revealed negative relationship of species diversity with biomass and carbon stocks. Furthermore, others studies found such relationships nonsignificant (see Gairola et al., 2011). These controversial outcomes suggest that the effects of diversity on forest carbon may vary with other factors such as the types and the successional stages of the forests (Lasky et al., 2014; Wu et al., 2015), and also the specific dimension of the diversity measure used (Con et al., 2013; Lasky et al., 2014; Ouyang et al., 2016).

### 4.3 Diversity effects mediated through functional diversity and functional dominance

The use of multiple diversity measures to provide additional insights into the mechanisms behind diversity–productivity has gained increasing interest in recent years (Cadotte et al., 2011; Conti & Díaz, 2013; Finegan et al., 2015; Lasky et al., 2014; Ruiz‐Benito et al., 2014; Vance‐Chalcraft, Willig, Cox, Lugo, & Scatena, 2010; Ziter, Bennett, & Gonzalez, 2013). Accordingly, functional diversity and dominance metrics were also examined in this study. While most of these studies tended to compare the relative effects of species richness and other diversity measures, we have provided here an additional example of exploring diversity effects on carbon stocks, by assuming that these effects were mediated through functional diversity and functional dominance. Our results on the structural equation modeling confirm this assumption and therefore support the need to explore beyond species richness to better elucidate the mechanisms that govern diversity–productivity relationship. The results further support the idea that both complementarity and selection effects are not exclusively affecting carbon storage (Ruiz‐Benito et al., 2014; Wu et al., 2015). Diversity (species richness) promotes carbon stock through effects of both functional diversity and functional dominance, partly because these diversity components are based on specific functional traits, which would reflect functional differences among the species (Díaz & Cabido, 2001; Song et al., 2014). This finding can also be due to the fact that increased species richness indirectly accounted for differences among species, in terms of ecological niche and resource use.

### 4.4 Functional diversity effects on tree carbon storage

Of the five functional diversity indices used in this study, only functional richness and functional evenness were found to explain variation in carbon stock. There is a variety of evidence for functional diversity effects on biomass and carbon. A study by Finegan et al. (2015) in tropical rain forests of Bolivia, Brazil, and Costa Rica found only functional richness—among other functional diversity indices—as significant predictor for biomass variation. Yet, a study in unmanaged forest fragments in Quebec revealed significant and positive relationships between functional dispersion and AGC (Ziter et al., 2013). Similarly, Ouyang et al. (2016) found significant but negative effects of the Rao quadratic entropy on stand biomass in subtropical forests in China. While we believe that these functional diversity indices have their specific biological meaning, in this study, the positive effect of functional richness on the AGC could be due to functional richness being positively correlated with species richness (SEM results; Villéger et al., 2008).

The functional richness measures the amount of trait or niche space filled by the species within a community (Clark et al., 2012; Mason, Mouillot, Lee, & Wilson, 2005). It would increase carbon storage because species with various traits would differ in resource use, and would more efficiently use the resources available within the community for higher growth and productivity, thus reflecting the niche complementarity effects (Finegan et al., 2015). Unlike functional richness, functional evenness did not show any relationship with species diversity; however, it did exhibit negative influence on AGC. Following Mason et al. (2005), the functional evenness measures the evenness of abundance distribution in the filled niche space. Therefore, both functional richness and functional evenness relate to the niche space or sections of niche space, and functional diversity as measured here could reflect some form of “niche differences” (Carroll, Cardinale, & Nisbet, 2011). Greater functional diversity, that is, greater value and range of functional traits, would reflect not only the magnitude of “niche differences”, but also the differences in resource utilization by species, thus promoting diversity effects on ecosystem functioning. This is in line with Carroll et al. (2011) who showed that increasing niche difference contributes to species coexistence and positive diversity effects on biomass yield.

The unexpected lack of strong individual effect of functional richness on aboveground carbon in this study might be due to the number of functional traits used. In fact, only three functional traits were considered; although these traits were found to be crucial to explain biomass allocation patterns (Chave et al., 2009; Mensah, Glèlè Kakaï, et al., 2016), they might not be as important as we thought for complementary resource allocation. Similarly, these functional traits might not be sufficient enough to catch the entire variability needed to explain carbon variation. Adding other functional traits such as plant hydraulic conductivity, leaf mass per area, and nitrogen fixing potential could have well captured the functional variability.

### 4.5 Functional dominance effects on tree carbon storage

The use of CWM values of functional trait to predict functional dominance effects is supported by the understanding that CWM metric reflects dominance of traits and species within a given community, and also in line with the fact that dominant species would induce functional shifts in mean trait values (Ricotta & Moretti, 2011). CWM as functional dominance metric could be used to elaborate on competitive dominance of species (Ricotta & Moretti, 2011). Therefore, functional dominance could indicate some aspect of “relative fitness differences” between competitors (Carroll & Nisbet, 2015; Carroll et al., 2011). Moreover, the finding that functional dominance significantly influenced tree carbon storage is consistent with the previous report that the magnitude of “relative fitness differences” strengthens the influence of diversity on biomass yield (Carroll et al., 2011). The functional dominance effects, as measured in this study, varied with the functional trait. Specifically, CWM of wood density revealed negative and significant effect on carbon stocks. It is not surprising given that wood density is a potential predictor of tree biomass, which highly correlates with the carbon stock. There are some insights that CWM of wood density is negatively related to the biomass increment, as being good predictor of individual tree diameter increments (Finegan et al., 2015). However, after examining biomass stocks in tropical forests, Stegen, Swenson, Valencia, Enquist, and Thompson (2009) pointed out that increasing wood density can decrease or increase the carbon stock, regardless of whether trees have high or low mean wood density. The authors therefore came to the conclusion that no general relationship exists between forest biomass and wood density. The present finding about CWM of wood density means that low wood density species grow faster and tend to store more biomass; thus, it suggests that conserving and planting low wood density species would likely help to increase the carbon stock.

Similarly, CWM of specific leaf area exhibited negative and significant effect on carbon stocks. This is consistent with other studies that found negative relationship between specific leaf area and plant biomass (Finegan et al., 2015). Leaf area is important for the amount of radiant energy intercepted by the plant. It is also generally known to facilitate the transfer of CO_2_ and water between foliage and atmosphere. Therefore, the significant influence of CWM of specific leaf area in this study supports the idea that leaf area captures a strategy of the plant for resource consumption, especially light (Mensah, Glèlè Kakaï, et al., 2016).

Community weight mean of maximum plant height showed positive relationship with carbon storage, as also reported in recent studies (Conti & Díaz, 2013; Finegan et al., 2015; Ruiz‐Jaen & Potvin, 2011). This is probably because tree height is a key variable for species‐specific or multispecies biomass regressions. In addition, maximal tree height is a potential species trait, as it defines the limits of competition for light and thus for light consumption (Poorter, Bongers, & Bongers, 2006; Poorter, Bongers, Sterck, & Woll, 2005). Examination of combined effects of functional dominance metrics revealed that only CWM of wood density and of maximum plant height were retained in the final model, with maximum plant height being the most significant predictor. Furthermore, only maximum plant height was also retained among functional dominance metrics when we assessed the combined effects of functional dominance and functional diversity. Tree height being closely related to tree diameter, the positive and significant relationship between CWM of maximum plant height and carbon stocks reflects the potential importance of characteristics of dominant and adult trees for ecosystem functioning and productivity, thus supporting the selection effects hypothesis. The important contribution of dominant stems to forest biomass has well been evidenced in some recent studies (Chave et al., 2003; Lung & Espira, 2015). The study by Lung and Espira (2015) revealed that tree stems larger than 50 cm have the greatest impact on forest biomass, and <16% of the species pool accounted for over 62% of the aboveground biomass.

### 4.6 Functional diversity effects greater than those of functional dominance

When examining the percentage of variance explained, we found that functional diversity explained more variance than functional dominance (Tables 2 and 4). This rejects our second hypothesis, and suggests that complementarity effects seem to be more important than selection effects. This finding contradicts Finegan et al.'s (2015) and Ruiz‐Jaen and Potvin's (2011) results that selection effects were more important for the aboveground biomass and carbon stock in tropical forests. For this study, functional dominance metrics (community weight mean of functional traits) were calculated using species relative abundance, while Ruiz‐Jaen and Potvin (2011) and Finegan et al. (2015) used species relative basal area and species relative biomass, respectively, as weighting variable. The strength of relationship between community weight mean of traits and the ecosystem function of interest could depend on the weighting variable. Biomass‐ or basal area‐weighted communities mean values would likely show stronger relation with biomass and carbon than abundance‐based communities mean values. Further studies should elaborate on this and show the extent to which weighting variable can influence our understanding of weighted mean values’ effects on ecosystem functions.

All being considered, it is important to mention that our result actually supports the idea that these two hypotheses (complementarity and selection effects) are not exclusive, and can contribute to ecosystem functioning. Previous evidence of both complementarity and selection effects on ecosystem function suggests they can also contribute at different proportions at different times of ecosystem development (Fargione et al., 2007). Both complementarity and selection effects mutually promote species coexistence. As pointed out by Carroll et al. (2011), these two hypotheses could even be the outcome of interactions of the “relative fitness differences” and the “niche differences”, whereby some species’ populations could be suppressed by dominant competitors, to allow effective utilization of the available resources. The selection effects reported here are strongly transmitted through specific maximum plant height, which reflects the influence of dominant species and suggests a possible competitive exclusion in terms of utilization of resources (e.g., light). In multispecies, multistory natural forests, chances are high to observe dominant and taller species that increase stand productivity, probably by achieving higher absorptivity of photosynthetically active radiation, thus reducing (through competitive dominance) the level of photosynthetic photon flux density available for understory species. However, it must be noted that, even for these dominant species, interactions within ontogenic stages (for example, competition for light between seedlings, juveniles, and adults) could define an efficient complementary use of light for greater productivity. Furthermore, an efficient use by understory species (limited to the subcanopy layer) of the available photosynthetic photon flux density, and also of decomposed litter (from canopy and dominant trees leaves) may likely reflect some complementary effects on stand productivity. Therefore, selection effects (dominant traits and species) on ecosystem function would be apparent in natural forests as we predicted, but complementary effects and efficient use of limited resources, especially by coexisting and understory species, could promote greater ecosystem function.

## 5 Conclusion

This study examined the diversity–carbon stock relationship in mistbelt forests in South Africa and revealed that taxonomic diversity (species richness) promotes carbon storage through functional diversity and functional dominance. The study further highlighted that both the niche complementarity and selection hypotheses are important for carbon storage. However, the effects of functional diversity (niche complementarity effects) were greater than functional dominance effects (selection effects). Moreover, the effects of functional dominance were strongly transmitted through the CWM of maximum plant height, reflecting the importance of forest vertical stratification for diversity–carbon relationship. Therefore, complementary effects would be induced also by complementary light‐use efficiency of species and trees growing in the understory layer. We suggest that future research on the relation between diversity and forest carbon be oriented toward a perspective of forest canopy (or dominant species vs. other species), to contribute additional insights into our understanding of biodiversity–ecosystem function relationship.
